# Safety and efficiency of peanut oral immunotherapy in preschool children with slow up-dosing and low maintenance dosing: a randomised controlled trial

**DOI:** 10.1016/j.lanepe.2026.101690

**Published:** 2026-05-06

**Authors:** Susanna Klevebro, Carina Uhl, Jon Roald Konradsen, Josefin Ullberg, Sandra Ganrud Tedner, Idun Holmdahl, Isabella Badolati, Rui Da Silva Rodrigues, Eva Sverremark-Ekström, Caroline Nilsson, Anna Asarnoj

**Affiliations:** a Department of Clinical Science and Education, Södersjukhuset, Karolinska Institutet, Stockholm, Sweden; b Paediatric Allergy and Pulmonology Unit at Sachs' Children and Youth Hospital, Stockholm, Sweden; c Department of Women's and Children's Health, Karolinska Institutet, Stockholm, Sweden; d Paediatric Allergy and Pulmonology Unit at Astrid Lindgren Children's Hospital, Karolinska University Hospital, Stockholm, Sweden; e Centre for Innovation, Research and Education, Region Västmanland, Västerås, Sweden; f Department of Molecular Biosciences, The Wenner-Gren Institute, Stockholm University, Stockholm, Sweden; g Department of Medicine, Solna, Karolinska Institutet, Stockholm, Sweden; h Department of Clinical Immunology and Transfusion Medicine, Karolinska University Hospital, Stockholm, Sweden

**Keywords:** Peanut allergy, Oral immunotherapy, Food allergy, Treatment, Ara h 2, Toddlers, Oral peanut challenge

## Abstract

**Background:**

Oral immunotherapy (OIT) is an emerging treatment for peanut allergy. A variety of protocols have been investigated, and accumulating evidence suggests that younger children may benefit from greater immune plasticity. The aim of the Small Children Oral Immunotherapy (SMACHO) study was to investigate the efficacy and safety of 3 years of peanut OIT with slow up-dosing and a low maintenance dose followed by 4–6 weeks of peanut-free diet in peanut-allergic toddlers.

**Methods:**

SMACHO is an open-labelled randomised controlled trial in Stockholm, Sweden (NCT04511494). After a positive peanut challenge to a cumulative dose of maximum 278 mg peanut protein, 75 allergic children, aged 1–3 years, were randomised 2:1 to peanut OIT, with slow up-dosing every 4–6 weeks and a low maintenance dose of 285 mg peanut protein, or to avoidance. Primary outcome was the proportion of children achieving sustained unresponsiveness, defined as tolerating a cumulative dose of ≥750 mg peanut protein in a challenge after 3 years of OIT followed by 4–6 weeks of a peanut-free diet.

**Findings:**

After OIT, 82% (41 of 50) achieved sustained unresponsiveness to a cumulative dose of ≥750 mg peanut protein. Before the peanut-free period, 84% (42 of 50) tolerated ≥750 mg peanut protein, compared with 3 of 25 children (12%) without treatment: difference 72% (95% confidence interval (CI) 56–88), p < 0·0001. Median cumulative tolerated dose after treatment was 5000 mg peanut protein compared with 3 mg after 3 years of peanut avoidance: difference 4997 mg (95% CI 4867–5127), p < 0·0001. Adverse events occurred in 0·7% of administered peanut doses, and most were mild. Six children reported eight severe dose-related events, with affected breathing, affected general well-being, or anaphylaxis. Epinephrine was administered at home three times in two children for dose-related reactions, all during up-dosing.

**Interpretation:**

Peanut OIT for young children, using slow up-dosing and a low maintenance dose, may be safer than protocols that escalate more rapidly or involve a higher maintenance dose. Our protocol can be implemented in clinical practice. When combined with early dietary introduction of peanut; this strategy has potential to contribute to a future decline in the prevalence of peanut allergy.

**Funding:**

This work was supported by a private non-commercial donation through Karolinska Institutet. Additionally by the Swedish Asthma and Allergy Association's Research Foundation; the Ellen, Walter and Lennart Hesselman Foundation for Scientific Research; Karolinska Institutet; Region Stockholm (ALF (FoUI-961605 and FoUI-973325) and clinical research appointments for Asarnoj, Nilsson, Konradsen and Klevebro); HRH Crown Princess Lovisa's association for child healthcare; the Samariten foundation for paediatric research; the Swedish Association for Allergology; the Freemasons of Sweden; the Swedish Society of Medicine; the Swedish Paediatric Society's Section for Allergy and Asthma, the Swedish Research Council (Dnr 2020-01839; 2023–02616); the Cancer and Allergy Foundation; the association Mjölkdroppen in Sweden; the Golden Jubilee Memorial Foundation; the Magnus Bergvall Foundation.

**Research in context**

**Evidence before this study**

Immunotherapy for airborne allergens has been practiced for more than 100 years. One form of immunotherapy, oral immunotherapy (OIT), has been investigated to a limited extent for food allergy, primarily in peanut allergy. The first publication addressing OIT was published in The Lancet in 1908.

To assess the existing evidence, we conducted PubMed searches for peer-reviewed papers published from inception to April 14 2026 using the search terms “immunotherapy”, “oral immunotherapy”, “peanut allergy”, “immunotherapy AND peanut allergy”, “oral immunotherapy AND peanut allergy”, and “oral immunotherapy AND safety”.

Previous trials have shown that OIT induces desensitisation but is associated with a substantial risk of adverse events, including severe allergic reactions. A large international multi-center trial of approximately 550 participants—most of whom were aged 4–17 years—showed a trend towards better outcomes in younger participants. A meta-analysis evaluating the safety of peanut OIT demonstrated that children aged 4–17 years undergoing OIT experienced an increased risk of anaphylaxis compared with untreated children.

A systematic review including 15 randomised controlled trials including 1530 participants, three of which enrolled children younger than 4 years, found that OIT increased desensitisation but was associated with higher rates of gastrointestinal adverse events and epinephrine use. In a separate study, 67% of families of peanut allergic infants or toddler's parents were asked about their interest in peanut OIT, indicating strong parental interest in early treatment despite limited evidence in this age group. One study evaluated remission rates and quality-adjusted life years (QALYs) gained in 201 children undergoing peanut OIT and assessed costs from a health-care payer perspective. The study found that peanut OIT was cost-effective compared with no treatment and was associated with improvements in quality of life.

We identified some trials in toddlers that assessed peanut OIT using alternative maintenance doses and varying durations of peanut avoidance after treatment cessation. Outcomes did not differ by maintenance dose; however, longer avoidance periods were associated with poorer outcomes, with fewer children maintaining tolerance. Trials in toddlers reported fewer severe adverse events than those typically observed in older children, although evidence regarding the safety and efficacy of peanut oral immunotherapy in early childhood remains limited.

To our knowledge, there is no evidence that describes peanut OIT in toddlers using the strategy of slow up-dosing and low maintenance dose.

**Added value of this study**

The SMACHO trial provides evidence that peanut oral immunotherapy (OIT) can achieve a favourable balance between efficacy and safety in preschool-aged children. In contrast with previous OIT studies, our protocol was associated with a markedly lower rate of severe adverse events while maintaining robust clinical outcomes. This was achieved by initiating treatment early in life and combining gradual up-dosing with a low maintenance dose administered every 4–6 weeks.

Our findings show that peanut OIT can be both highly effective and substantially safer than previously reported approaches. After three years of treatment followed by a 4–6-week avoidance period, almost all participants remained tolerant to peanut. Sustained unresponsiveness (SU) to 750 mg peanut protein was achieved in 82% of the intention-to-treat population and 98% of the per-protocol population. Among participants completing the protocol, 35 of 42 tolerated 5000 mg peanut protein during the SU oral food challenge. Adverse events were predominantly mild, and 22% of children experienced no adverse events at all. The minimal use of home-administered adrenaline further indicates an exceptionally favourable safety profile.

**Implications of all the available evidence**

Our findings indicate that initiating peanut OIT in early childhood, combined with gradual dose escalation and a low maintenance regimen, can minimise adverse events while preserving therapeutic efficacy. This approach addresses key evidence gaps related to age-specific immune responsiveness and the need for better tolerability in routine clinical practice. Importantly, the extended intervals between up-dosing visits reduced treatment burden, facilitated family adherence, and enhanced feasibility. The high palatability of study doses, reported by most participants, likely contributed to excellent compliance and overall treatment success. Taken together, these results suggest that a low-intensity, early-age OIT strategy could provide a practical and effective opportunity to induce remission of peanut allergy and reduce the risk of severe allergic reactions later in life.

## Introduction

Peanut allergy has increased globally and affects about two percent of the population in Western Europe and North America. It is often lifelong; only 20–30% outgrow their allergy.^1^ Oral immunotherapy (OIT), in which a small amount of the allergenic food is given regularly, has emerged as a treatment method.^2^ The dose is gradually increased until a maintenance dose is reached and continued for a specified time.^3^, ^4^, ^5^, ^6^, ^7^ OIT can improve the quality of life of the allergic individual.^5^ However, adverse reactions during OIT are very common; severe reactions occur in up to 20% but decrease with longer treatment.^5^^,^^7^^,^^8^ High maintenance doses in OIT increase the percentage of reactions requiring treatment with epinephrine, without evidence of greater tolerance.^9^ Most studies involve children over 4 years, but younger age seems linked to better safety and fewer adverse reactions.^3^^,^^4^^,^^10^, ^11^, ^12^, ^13^ Dose-escalation strategies may also influence the frequency of adverse events. Most peanut OIT studies have used protocols with biweekly up-dosing,^2^, ^3^, ^4^^,^^7^^,^^8^^,^^14^, ^15^, ^16^ whereas some Asian OIT studies involving staple foods applied a slower escalation strategy,^17^^,^^18^ which may result in fewer adverse events.^19^

OIT induces tolerance to allergens, typically referred to as desensitisation: tolerance during continuous exposure.^4^^,^^8^^,^^16^ Some studies assess sustained unresponsiveness (SU), defined as the persistence of efficacy after withdrawal of treatment,^3^^,^^6^^,^^20^ which may reflect everyday clinical tolerance more accurately than desensitisation.

We designed the Small Children Oral Immunotherapy (SMACHO) trial to build upon prior clinical experience and to evaluate whether a slower up-dosing regimen combined with a lower maintenance dose could maintain efficacy, including SU, while improving the adverse event profile. This randomised controlled trial (RCT) enrolled preschool children aged 12–47 months with challenge-confirmed peanut allergy. Participants were randomised to peanut avoidance or OIT with slow up-dosing every 4–6 weeks to a low maintenance dose of 285 mg peanut protein. We have previously presented interim data, from the 1-year oral peanut challenge (OPC), demonstrating that 72% in the OIT group achieved the predefined outcome, compared with 4% in the Avoidance group.^21^ The children on OIT tolerated a median cumulative dose of 3500 mg (interquartile range [IQR] 1750–5000) peanut protein compared with 2·8 mg (IQR 2·6–27·8) in the Avoidance group.^21^

Immunological processes occurring during OIT include an initial rise in allergen-specific immunoglobulin E antibodies (IgE ab), followed by a subsequent decline, considered indicative of therapeutic efficacy.^22^^,^^23^ In contrast, allergen-specific immunoglobulin G4 antibodies (IgG4 ab) typically increase as IgE ab levels decrease.^23^ To date, no additional biomarkers have been identified that reliably predict treatment success or the likelihood of adverse reactions in OIT.

The aim of SMACHO was to evaluate the efficacy and safety of 3 years of peanut OIT with slow up-dosing and a low maintenance dose followed by 4–6 weeks of peanut-free diet in peanut allergic preschool children. We report the clinical and immunological results after 3 years of peanut OIT and 4–6 weeks of a strict peanut-free diet.

## Methods

### Study design

SMACHO is a clinical open-labelled RCT, conducted at the research clinic of the Sachs’ Children and Youth Hospital, Södersjukhuset, in Stockholm, Sweden.^24^ The study was approved by the Swedish Ethical Review Authority (2019–04645, 2020–04559, 2022–040107-02, 2023-05159-01, 2024-02068-02, and 2024-07975-02) and registered on ClinicalTrials.gov, NCT04511494.

### Participants

Children in Stockholm, aged 12–47 months, with IgE ab to whole peanut extract or *Arachis hypogaea* 2 (Ara h 2) over 0·1 kU_A_/L, were identified through the Karolinska University Hospital Laboratory, which performs routine testing on samples from various levels of care. A total of 996 children met these requirements and 289 were randomly selected and invited to participate. Caregivers of 84 children accepted the invitation and signed a written consent form. The children attended a clinical visit during which an allergy-focused history, including any previous reactions to peanut, was obtained. Their general medical history, encompassing current and past medications, was reviewed, and a physical examination was conducted.

Exclusion criteria were other serious illness, previous life-threatening anaphylaxis (intensive care), a history of eosinophilic esophagitis or other gastrointestinal disorders, severe uncontrolled asthma, or ongoing medication with biological drugs or oral steroids. Provided that no exclusion criterion was fulfilled, the child was included in the study and underwent a baseline OPC before randomisation. Sex was reported by parents, and slightly more boys were randomised to avoidance (see Table 1).

**Table 1:** Characteristics of the study participants at baseline and three-year follow-up.

	OIT		OIT		Avoidance		Avoidance	
	Baseline, n = 50		3 years, n = 42		Baseline, n = 25		3 years, n = 20	
	Median	IQR	Median	IQR	Median	IQR	Median	IQR
Age (months)	30·5	23·0–40·0	68·0	60·0–77·0	31·0	24·0–38·0	67·0	59·5–77·0
Baseline dose provokinga reaction (mg)	215	28–278			278	28–278		
IgE ab to peanut (kUA/L)	5·2^a^	0·9–31·5	0·9^b^	0·1–5·6	25·2^c^	2·9–74·3	33·6^d^	1·3–173·0
IgE ab to Ara h 2 (kU_A_/L)	4·4^e^	0·7–16·8	0·5^f^	0·04–2·4	7·5^g^	2·7–42·5	14·2^h^	0·8–89·4
	n	%	n	%	n	%	n	%

Female	27/50	54%			10/25	40%		
Any parent allergic	44/50	88%			21/25	84%		
Allergic to milk, egg, or wheat	29/50	58%	8/42	19%	12/25	48%	7/20	35%
Atopic dermatitis	43/50	86%	26/41	63%	22/25	88%	9/20	45%
Rhinoconjunctivitis	12/50	24%	23/42	55%	8/25	32%	7/19	37%
Asthma/wheeze	19/50	38%	20/41	49%	7/25	28%	12/19	63%

OIT denotes oral immunotherapy; IQR denotes interquartile range; IgE denotes immunoglobulin E; ab denotes antibodies.

Ara h 2 denotes Arachis hypogaea 2.

a n = 47.

b n = 35.

c n = 23.

d n = 19.

e n = 49.

f n = 35.

g n = 24.

h n = 19.

### Randomisation and masking

The study team used STATA program version 15·1 to generate a random 2:1 allocation sequence with block randomisation. The list with the allocation sequence was given to an administrator outside the project, to conceal it from the study team. The administrator prepared numbered, sealed, opaque envelopes containing study number and allocation group. The envelopes were opened in numerical order by the doctor together with families, one at a time, after positive OPCs. No post-randomisation masking occurred.

### Procedures

**Fig. 1:**
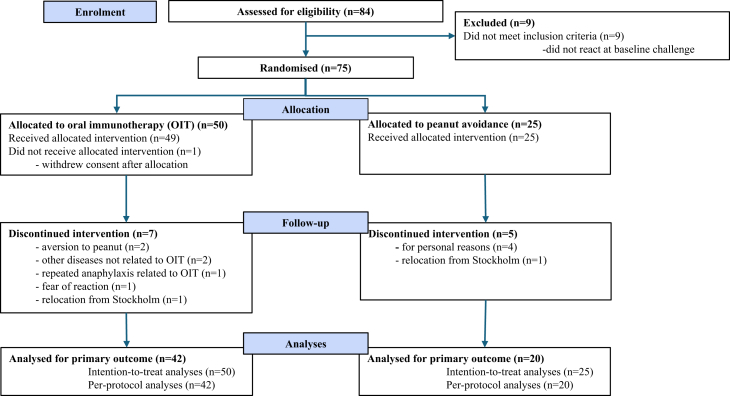
**Flowchart of participants included in the study (n = 75), with reasons for exclusion at each stage, and the final number of individuals included in the primary outcome analysis (n = 62)**.

The study protocol has previously been described in detail.^24^ The baseline OPC was performed with increasing doses (0·3, 2·5, 25, and 250 mg peanut protein), delivered in the form of peanut butter (Garant, Axfood AB, the Netherlands, 99·3% peanut 0·7% salt), at 30-min intervals and was stopped if symptoms occurred. Children with at least one objective allergic symptom, eg, lip swelling or a rash, after a maximal cumulative dose of 277·8 mg peanut protein, were assessed as having a positive OPC and were randomised 2:1 to OIT or peanut avoidance (Fig. 1). Nine children without reaction at baseline OPC were not eligible for randomisation.

Children randomised to OIT initiated treatment with a peanut dose equivalent to approximately 1/4 of their individual reaction dose established at the baseline OPC, up to a maximum of 48 mg peanut protein. This starting dose was administered in the clinic as a four-step mini-challenge, with the final step corresponding to 1/4 of each child's reaction dose. Doses up to 24 mg peanut protein were administered as a mixture of peanut flour (peanøttmel, 50% protein content, Sukrin, Oslo, Norway) and oat flour (4·6% peanut protein), yielding a final mixture containing 4·6% peanut protein. The research nurses at the Research Unit dispensed the flour mixture into individual daily containers, which parents took home for at-home dosing.^24^ All other doses were given with increasing amounts of peanut-containing corn puffs (BAMBA, Osem Food Industries, Israel) with 95 mg peanut protein in each.^25^ Children visited the hospital every 4–6 weeks for dose escalation. Between scheduled visits, the children continued to take the tolerated dose at home on a daily basis. When the maintenance dose of 285 mg peanut protein (three BAMBAs) was reached, children maintained this dose during the treatment period, with clinical visits every third month. The peanut dose and time of intake along with adverse events or concomitant medications were assessed through daily reports and discussed with the staff at each clinical visit, for safety reason. The Avoidance group completed clinic-based follow-up evaluations at the 1-year and 3-year timepoints. Between visits, they reported any accidental peanut ingestion on a monthly basis and were able to contact the Research Unit for advice as needed. After 1 and 3 years, the OIT group performed an OPC with up to six doses, 250, 500, 1000, 1000, 1000, and 1250 mg peanut protein at 30-min intervals, for a maximal cumulative dose of 5000 mg peanut protein. After 3 years of treatment and 4–6 weeks of peanut-free diet, another OPC with the same doses was performed to assess sustained unresponsiveness (SU). The Avoidance group repeated the OPC performed at baseline after 1 and 3 years of avoidance and continued with additional doses, like the OIT group, if they had no reaction at lower doses.

Classification of the intensity of an adverse event was defined as follows: Mild events were transient or easily tolerated and did not significantly interfere with daily activities; examples include oral itching. Moderate events caused discomfort and interfered with daily activities and might require minimal intervention; examples include mild asthma responding to treatment or gastrointestinal symptoms. Severe events caused significant interference with daily life and required medical intervention or hospitalization; examples include anaphylaxis or breathing difficulties.

Blood samples were drawn at the time of OPCs. For immunological analyses, 5 mL of whole blood was drawn into an EDTA tube. Following collection, the tube was kept at room temperature and subsequently centrifuged at 2000×*g* (equivalent to 3993 rpm) for 10 min to separate plasma from blood cells. The aliquoted plasma was stored at −80 °C pending analysis. ImmunoCAP (Thermo Fisher Scientific, Uppsala, Sweden) was used to analyse IgE ab and IgG4 ab to whole peanut extract, Ara h 1, Ara h 2, Ara h 3, and Ara h 6.

### Outcomes

The primary outcome was the efficacy of peanut OIT using a slow up-dosing regimen and a low maintenance dose, measured as the proportion of children achieving SU, defined as tolerating a cumulative dose of ≥750 mg peanut protein during an OPC after 3 years of OIT followed by 4–6 weeks of a peanut-free diet. Outcomes from the 3-year OPC and the SU OPC in the OIT group were compared with outcomes from the 3-year OPC in the Avoidance group. Secondary outcomes included desensitisation to ≥750 mg of peanut protein during the OPC after 3 years of OIT, frequency of adverse events, and changes in immunological parameters (IgE ab and IgG4 ab levels) measured after 1 year and at the final OPCs. Additional post hoc outcomes were tolerating a cumulative dose of 5000 mg peanut protein during the OPC after 3 years of OIT and the SU OPC, as well as differences in allergic co-morbidities as specified in the Supplement. We also report descriptive data on the severity of allergic reactions during the final OPCs and adherence. The Food Allergy Severity Score (FASS) was used to assess the severity of allergic reactions during OPC.^26^

### Statistical analysis

A sample size calculation with 80% power and a two-sided 5% significance level indicated the need for 30 individuals in the OIT group and 15 individuals in the Avoidance group to detect a 40% difference in the primary outcome between the groups. To account for potential attrition and considering secondary and exploratory outcomes, we increased the sample sizes to 50 and 25 individuals, respectively.

Stata version 18·0 was used for the analyses. Continuous non-normally distributed variables are presented as median with interquartile range (IQR; 25th–75th percentile). Logistic regression was used to analyse the primary outcome and to conduct post hoc analyses of the proportion of participants tolerating a cumulative dose of 5000 mg peanut protein. Risk difference with 95% confidence interval (CIs) were derived from the logistic model. Pre-specified analyses of effect modification by age and IgE ab to peanut and Ara h 2 at baseline were conducted by including an interaction term between treatment group and baseline variable in the logistic regression. Post-hoc analyses adjusting for IgE ab to peanut at baseline were performed as a sensitivity analysis due to baseline imbalances despite randomisation (21). Both intention-to-treat (ITT) and per-protocol (PP) analyses were performed. The ITT population consisted of all randomised children, with participants with missing outcome data assumed to be intolerant. The PP population consisted of all children completing the study (Fig. 1). Comparisons of medians were performed using quantile regression with bootstrapped CIs.

Values of IgE ab reported as <0·1 kU_A_/L were replaced by 0·0999 kU_A_/L. In the statistical analyses, values of IgE ab and IgG4 ab reported as 0 were replaced by 0·01. To analyse treatment effects on antibody levels over time, multilevel mixed-effects linear regression models with a time × treatment interaction were used with random intercepts and random slopes for follow-up time at the participant level and an unstructured covariance. Logarithmically transformed antibody levels were used to approximate normality of the residuals. The Mann–Whitney U test was used to compare cross-sectional differences in antibody levels. To analyse treatment effects on the prevalence of other allergic manifestations over time, multilevel mixed-effects logistic regression models with a time × treatment interaction were used, with participant identity included as a random-effects term.

There was no data monitoring committee established for this study.

### Role of the funding source

The role of the funders was limited to providing financial support. They had no influence on study design, data collection, data analysis, data interpretation, or writing of the report.

## Results

In total, 75 children were enrolled in the study between 16 September 2020 and 30 March 2022, with 50 children randomised to the OIT group and 25 to the Avoidance group, representing the ITT population (Fig. 1). Baseline characteristics have been reported previously and are shown in Table 1.^21^ In the OIT group, eight of 50 children (16%) discontinued the trial, one prior to initiating OIT, four before the 1-year follow-up, and two thereafter, one of whom never performed the 1-year challenge. In the Avoidance group, five of 25 children (20%) discontinued the trial, all prior to the 1-year follow-up (Fig. 1). Table S1 shows age, IgE ab to peanut and Ara h 2, and the history of previous allergic reactions to peanuts among children who discontinued the trial and those in the PP population. Children who discontinued the trial appeared to be older and had higher IgE ab levels, although these differences were not statistically assessed because of the small number of discontinuations.

**Fig. 2:**
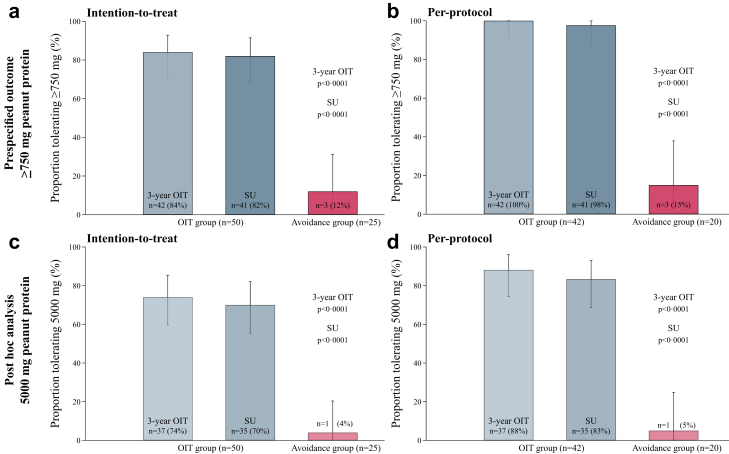
**Proportion of children who performed oral peanut challenges after 3 years of oral immunotherapy (OIT), after 3 years of OIT followed by 4**–**6 weeks of peanut avoidance (sustained unresponsiveness; SU), and after 3 years of peanut avoidance.** Panels A and B show the primary outcome: the proportion of children tolerating a cumulative dose of ≥750 mg peanut protein in the intention-to-treat (a) and per-protocol (b) populations. Panels c and d show the proportion of children tolerating a cumulative dose of 5000 mg peanut protein in the intention-to-treat (c) and per-protocol (d) populations.

For the prespecified primary outcome—tolerance at OPC after 3 years of OIT followed by a 4–6 weeks peanut-free period, compared with 3 years of avoidance—the risk difference between the OIT and Avoidance groups was 70% (95% CI 53–87; p < 0·0001; Fig. 2a). The median cumulative tolerated dose in the OIT group was 5000 mg (IQR 3750–5000), equivalent to approximately 25 peanuts. In contrast, the Avoidance group had a median tolerated dose of 3 mg (IQR 3–153), corresponding to a difference of 4997 mg (95% CI 4867–5127; p < 0·0001). The proportion of children tolerating 5000 mg is shown in Fig. 2c, with a risk difference of 66% (95% CI 51–81; p < 0·0001) compared with the Avoidance group. At the OPC after 3 years, before the peanut-free period in the OIT group, 42 of 50 (84%) children tolerated a cumulative dose of ≥750 mg peanut, a difference of 72% (95% CI 56–88; p < 0·0001) compared with the Avoidance group (Fig. 2a).

In the PP population, 41 of 42 children in the OIT group achieved SU to ≥750 mg peanut protein (Fig. 2b). The difference compared with the Avoidance group after 3 years was 83% (95% CI 66–99%; p < 0·0001). The median cumulative tolerated dose at the SU challenge after OIT was 5000 mg (IQR 5000–5000), compared with 28 mg (IQR 3–278) in the Avoidance group, p < 0·0001. The maximal cumulative dose of 5000 mg peanut protein was tolerated by 35 of 42 children in the OIT group at the SU OPC (Fig. 2d and Figure S1) and the risk difference compared with the Avoidance group was 78% (95% CI 64–93; p < 0·0001). At the 3-year OPC, prior to the peanut-free period, all participants in the OIT group tolerated the cumulative dose of ≥750 mg peanut protein (Fig. 2b). The maximal cumulative dose of 5000 mg peanut protein was tolerated by one child in the Avoidance group and 37 children in the OIT group (Fig. 2d), whereas five children tolerated doses above 750 mg but below 5000 mg: one child tolerated 2750 mg, and four children tolerated 3750 mg. The number of children tolerating each cumulative dose is shown in the Supplement (Figure S1).

The treatment effect on tolerating ≥750 mg of peanut protein was not modified by age or baseline IgE ab levels, and no significant effect modification was observed for the 5000 mg outcome either. However, these analyses should be interpreted with caution because of the small number of children in the OIT group who did not tolerate 750 mg and the small number in the Avoidance group who tolerated 5000 mg. Sensitivity analyses adjusting for baseline levels of IgE ab to peanut did not change the result, with an adjusted risk difference for the primary outcome in the ITT population of 70% (95% CI 52–87%; p < 0·0001).

**Fig. 3:**
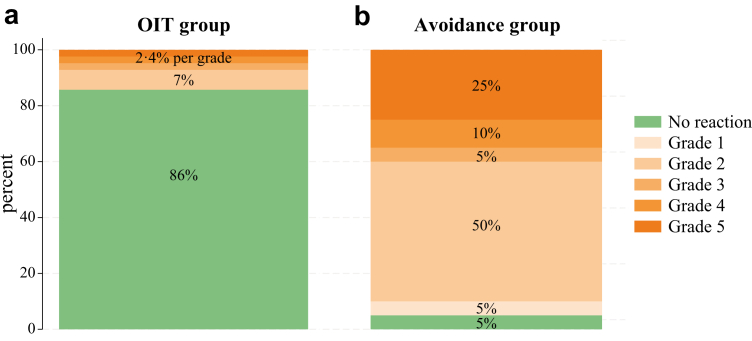
**Proportion of children by grade of severity of the allergic reaction as described by the Food Allergy Severity Score****^26^****at the a) oral peanut challenge (OPC) after 3 years of oral immunotherapy (OIT) and 4–6 weeks peanut-free diet in the OIT group and b) OPC after 3 years of strict peanut avoidance in the Avoidance group**.

The severity of the allergic reactions during OPC was compared between the Avoidance group, after 3 years of strict peanut-free diet, and the OIT group, after 3 years OIT and a subsequent peanut-free period of 4–6 weeks.^26^ Reactions were notably milder in the OIT group, with 2·4% experiencing grade five reactions, eg, anaphylaxis or breathing difficulties, compared with 25% in the Avoidance group (Fig. 3).

**Fig. 4:**
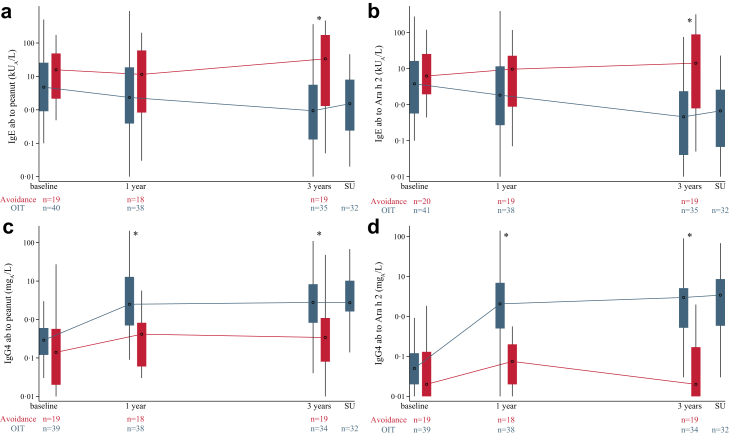
**Immune parameters at baseline, 1 year, 3 years, and at the sustained unresponsiveness (SU) evaluation (after 3 years of oral immunotherapy (OIT) and 4**–**6 weeks of peanut avoidance) in the per-protocol population, with the OIT group shown in blue and the Avoidance group in red.** Medians and interquartile ranges (IQRs) are displayed; asterisks indicate between-group differences at p < 0·05 (Mann–Whitney U test). Panels a and b show immunoglobulin E (IgE) antibodies to peanut (a) and Ara h 2 (b), both demonstrating significant time × treatment interactions (peanut p < 0·0001; Ara h 2 p = 0·007). Between-group differences were significant at the 3-year follow-up for both peanut-specific IgE (p = 0·0025) and Ara h 2-specific IgE (p = 0·0008), but not at 1 year (p = 0·16 and p = 0·17, respectively). Panels c and d show immunoglobulin G4 (IgG4) antibodies to peanut (c) and Ara h 2 (d), each with significant time × treatment interactions (peanut p = 0·006; Ara h 2 p = 0·003) and significant between-group differences at both 1 year (peanut p = 0·0004; Ara h 2 p < 0·0001) and 3 years (both p < 0·0001).

IgE ab levels to peanut and Ara h 2 did not differ between the OIT and Avoidance groups at baseline, although there was a tendency towards higher levels in the Avoidance group (p = 0·057 and p = 0·12, respectively). The three children in the Avoidance group who developed peanut tolerance had IgE ab to Ara h 2 of 0·66, 1·77, and 5·6 kU_A_/L, respectively, at baseline. There was a significant effect of treatment on trajectories of IgE ab to peanut and Ara h 2 over time (Fig. 4). IgE ab levels to peanut and Ara h 2 decreased over time during OIT and were significantly lower than in the Avoidance group after 3 years (Fig. 4a–b). No change in IgE ab levels was observed following the four-week peanut-free period in the OIT group. A significant time × treatment interaction was also observed for IgG4 levels. In the OIT group, IgG4 ab levels to peanut and Ara h 2 increased over time, with a significant between-group difference evident after 1 year (Fig. 4 c–d). No changes over time in IgG4 ab levels were detected in the Avoidance group.

No significant treatment effect was observed on the prevalence of other allergic manifestations from baseline to the 3-year follow-up. The children had lower prevalence of atopic dermatitis and milk, egg, or wheat allergy but higher prevalence of rhinoconjunctivitis and wheeze compared with at baseline. However, there were no between-group differences in prevalence of these co-morbidities, and the treatment had no significant effect on the changes in prevalence over time (Supplement, Figure S2).

A total of 43,537 doses were administered during the 3 years of peanut OIT. The median proportion of missed doses per participant over the treatment period was 4·2% (IQR 1·9–7·4). During the up-dosing phase, parents reported a median of 5·1% (IQR 2·0–8·5) missed doses and, during maintenance, the reported median was 3·5% (IQR 1·8–6·9). The median duration of dose escalation was 9 months (IQR 7·5–12·0).

Adverse events occurred after 0·7% of administered doses. In the ITT population, 11 of 50 (22%) children in the OIT group had no allergic reactions related to the daily peanut OIT dose during the complete treatment period of 3 years. Dose-related adverse events during OIT were most common in the first month but occurred in all study phases (Supplement, Figure S3). The median number of reported adverse events per participant was 3·5 events (range 0–33). During the up-dosing, children experienced a median of three events (range 0–28) per participant, whereas during the maintenance phase, the median was zero events (range 0–16) per participant. Eight of 42 children (19%) who completed 3 years of OIT reported no allergic reactions at all.

**Fig. 5:**
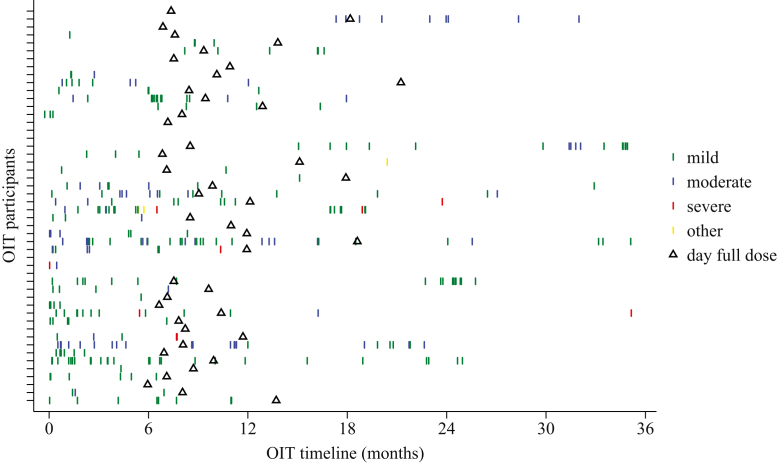
**Adverse events related to the daily peanut oral immunotherapy (OIT) dose among children receiving OIT during 3 years of treatment. Each row represents one participant, with lines coloured according to reaction severity. Black triangles indicate the initiation of the maintenance phase**.

The most frequently reported dose-related adverse events were mild (72%), involving oral or lip symptoms, urticaria, or eczema (Table S3). Gastrointestinal symptoms constituted 9% of all reported adverse events. None of the children had persistent gastric symptoms or symptoms indicative of eosinophilic esophagitis. Eight severe dose-related adverse events were reported in six children, defined as reactions including affected breathing, affected general well-being, or anaphylaxis. Six of these reactions occurred during up-dosing (Fig. 5). Four of the severe reactions were assessed as anaphylaxis; all occurred during up-dosing.

Dose-related epinephrine administration at home occurred three times during the trial.^21^ Two children in the OIT group administered epinephrine auto-injectors during the up-dosing phase and none during the maintenance phase. The participant requiring dose-related epinephrine administration twice withdrew from the study after 1 year of OIT before reaching the maintenance dose. Six children in the OIT group received seven doses of epinephrine during the study period due to allergic reactions after accidental intake of other foods than peanuts (milk, wheat, or tree nuts). Seven children in the Avoidance group experienced symptoms following accidental peanut exposure, each on a single occasion. Four reactions were classified as severe, involving respiratory or systemic symptoms. Two children received antihistamines only, four were treated with both antihistamines and oral corticosteroids, and none were treated with epinephrine.

## Discussion

The SMACHO peanut OIT study shows a favourable balance of efficacy and safety, supporting the strategy of young age, slow up-dosing, and low maintenance dose. Our study finds a high proportion of patients with SU to ≥750 mg peanut protein after 3 years of peanut OIT followed by 4–6 weeks of peanut-free diet (82% in ITT and 98% in PP), and relatively mild adverse events with few instances of home-administered epinephrine. To our knowledge, the incidence of adverse events demonstrated in this study is the lowest yet, in combination with a strong OIT treatment effect. Moreover, SMACHO is the first RCT evaluating low-dose peanut OIT with commercially available peanut puffs. Despite the low maintenance dose of 285 mg peanut protein, 83% of children in the PP population tolerated 5000 mg at the SU challenge, after 3 years of OIT and 4–6 weeks of a peanut free diet.

The treatment effect in our study was similar to or slightly better than those in other toddler OIT studies. The IMPACT study was a randomised, double-blind, placebo-controlled trial investigating the safety and efficacy of peanut OIT in young children, aged 12–47 months, with confirmed peanut allergy.^3^ The children received peanut OIT or placebo for 2·5 years, during which they were up-dosed every 2 weeks to reach a maintenance dose of 2000 mg of peanut protein per day followed by a 6-month avoidance period. After the treatment period, 71% were desensitised to 5000 mg peanut protein; in our study, that figure was 74%. In the phase 3 POSEIDON study, a randomised, double-blind, placebo-controlled trial investigating peanut OIT in toddlers (aged 1–4 years), with biweekly dose escalation intervals and a maintenance dose of 300 mg peanut protein but no SU evaluation, 73% tolerated 600 mg of peanut protein during the final peanut challenge following 1 year of treatment.^12^ However, it is unclear if the children tolerated a higher dose. The DEVIL study on peanut OIT in children (9–36 months), with biweekly up-dosing, compared SU between low and high maintenance doses.^4^ Following 3 years of peanut OIT and a subsequent 4-week peanut-free period, 78% achieved SU (both low and high dose), compared with 82% in our study. SU may be a more clinically relevant evaluation metric than desensitisation. However, few studies use this endpoint.^3^^,^^4^ Like SMACHO, the IMPACT and DEVIL studies evaluated outcome using both endpoints.

We evaluated SU following 4–6 weeks of peanut-free diet in our study, and the effect persisted in all but one of the children in the PP population, consistent with findings from the DEVIL study.^4^ In contrast, in the IMPACT study, with a six-month discontinuation of peanut consumption, only 21% retained their tolerance. The difference is most likely attributable to the prolonged avoidance period of 6 months in the IMPACT trial.^3^ In POISED, a study with older participants aged 7–55 years, 85% were desensitised to 4000 mg peanut protein after 2 years of OIT and 35% achieved SU after 3 months of avoidance but only 13% achieved SU after 1 year of avoidance.^6^ After the avoidance period in SMACHO, 35/43 children still tolerated the maximal cumulative dose of 5000 mg peanut protein, whereas seven tolerated lower doses: 250 mg (n = 1), 750 mg (n = 1), 2750 mg (n = 1), and 3750 mg (n = 4) (Supplement, Figure S1). These findings indicate that the duration of peanut avoidance strongly influences the outcome, with prolonged interruption periods being insufficient to maintain SU. In contrast, shorter interruptions—such as a 1-month pause during travel—may be feasible without affecting the treatment outcome. The study finished after the final OPC, and children were thereafter free to determine their own peanut intake. We advised children in SMACHO to continue consuming at least 1·5 peanuts at least three times per week, to maintain desensitisation. They were permitted to ingest larger amounts if they wished, provided that the quantity did not exceed the maximum dose tolerated during the final challenge.

When safety profiles and adverse events are evaluated across studies, notable differences emerge.^2^^,^^7^ In the IMPACT study, with participants of similar age as in our study but a high maintenance dose of 2000 mg peanut protein, treatment-related adverse events were reported in 98% of participants, compared with 78% in our study. Furthermore, 22% of participants in the IMPACT study required epinephrine treatment at home for therapy-related reactions, whereas the corresponding figure in our study was 4%, which may be due to milder adverse effects in our study. Also, in the POSEIDON and DEVIL studies, dose escalation was conducted biweekly. In the DEVIL study, 95% of subjects experienced adverse events likely related to OIT; in our study, 22% reported no adverse events. In the POSEIDON study, much like in ours, 75·5% experienced treatment-related adverse events after 1 year of OIT, compared with 73% in our study. Additionally, treatment-related epinephrine use at home was reported in 2% of participants of POSEIDON, compared with 4% in SMACHO.^12^ In the DEVIL study, comparisons between a lower (300 mg) and higher (3000 mg) maintenance dose of peanut protein revealed similar or slightly better SU in the group receiving 300 mg peanut protein daily.^4^ Taken together, data from other studies along with our own findings may support the use of a lower treatment dose of around 300 mg peanut protein. Our study reports a lower incidence of adverse events, which may be explained by the low patient age, the slower dose escalation, and the low maintenance dose. These factors may collectively contribute to a favourable safety profile while maintaining efficacy.

SMACHO is the first randomised peanut OIT trial using a slow up-dosing strategy with dose escalation every 4–6 weeks. The rationale for this strategy was to assess the frequency and severity of adverse events during peanut OIT. The other known peanut OIT studies for young children, IMPACT, POSEIDON, and DEVIL, all implemented up-dosing in biweekly intervals. However, some Japanese studies have explored a protocol in children with food allergy in which patients undergo an oral food challenge, then continue regular intake below their reaction threshold. This maintenance dose is administered over several months, for up to 1 year.^17^^,^^18^^,^^27^ Most children tolerate a higher dose than initially at follow-up challenge.^28^ Our slow up-dosing strategy (every 4–6 weeks) does not seem to have any disadvantages compared with standard intervals of 2 weeks. A clear advantage with our long and flexible dose escalation intervals was the feasibility for participating families, with easier adaptation to infections, travel, etc., factors that we believe increased the commitment to the protocol and reduced dropout frequency. Further research on extended dosing intervals in peanut OIT would be desirable.

Another feature unique to SMACHO was the usage of the well-characterised and commercially available peanut puffs BAMBA for doses over 24 mg.^21^ The peanut puffs were well-tolerated by children undergoing OIT in the study, with respect to both palatability and texture. BAMBA also carry a low risk of choking, as they dissolve in contact with saliva. They were used in the LEAP study^29^ and in a real-world OIT study.^10^^,^^11^ SMACHO is the first RCT confirming that the usage of BAMBA is safe and effective, despite the inevitable variation in daily ingested doses due to variation in puff size and cutting of puffs during the up-dosing phase. Consequently, peanut OIT can be carried out in a cheap, safe, and effective way. In SMACHO, a mixture of oat and peanut flour was used for doses up to 24 mg of peanut protein to permit accurate and reproducible administration of very low dose levels. The use of a standardised peanut OIT drug could substantially streamline dosing at these lower thresholds, as the manual preparation and weighing of such small quantities is time-consuming and may introduce avoidable variability.

The IMPACT and POSEIDON studies both showed an age gradient towards better effect. In IMPACT, there were higher rates of SU in children below 2 years of age at inclusion. Additionally, both IMPACT and DEVIL found inverse relationships between peanut IgE ab and outcome success. In SMACHO, we did not see such associations with either age or peanut IgE ab at baseline, possibly due to insufficient statistical power: too few children in the younger age span and few children who failed the final challenge in the treatment group.

Another area of interest is the potential impact of peanut OIT on the development of other allergic manifestations. However, no differences were observed between the peanut OIT and Avoidance groups from baseline to endpoint. Neither OIT nor preventive treatment with peanuts have been shown to impact the development of allergic comorbidities in other studies.^6^^,^^30^

The main strengths of this study were the high feasibility, with long and flexible dose escalation intervals, and the treatment with peanut puffs, which the participating children liked to eat. These factors made the participants and their families satisfied and compliant with the study protocol. The main weakness of the study was its open label design without a placebo group, due to the challenges of masking peanut puffs as placebo. Consequently, the intervention was not blinded to the participants or study staff, and comparisons with placebo-controlled peanut OIT trials should therefore be made with caution. A further methodological limitation was that the presence of a single objective sign during the baseline challenge was sufficient for randomisation. These manifestations could be mild, such as skin reactions including lip swelling. Still, 30% of the randomised children required administration of epinephrine during the baseline OPC. Additional weaknesses included the absence of blinded food challenges, and the fact that the follow-up challenges after 1 and 3 years were conducted with different starting doses for the OIT and Avoidance groups. A recent study found that double-blind placebo-controlled food challenges offer no advantage over open food challenges when performed by trained staff using strict objective stopping criteria.^31^ The differing starting doses were justified by the fact that it was known (not blinded) that participants in the OIT group were already ingesting 285 mg peanut protein daily; thus, testing with lower doses was deemed unnecessary. The relatively small sample size of 75 participants constitutes an additional limitation that may restrict the generalisability of the findings. Furthermore, because adverse events were parent-reported, there is a potential risk of measurement error. The generalisability of the findings is limited to younger children sensitised to peanut; the findings would probably differ for older children and adults. However, the study included children from both primary care and specialist care settings, and children with both mild and severe peanut allergy at baseline.

There is a need for further research, particularly head-to-head trials comparing different OIT protocols. Variations in dose, treatment duration, and build-up strategies may affect both efficacy and safety; however, these parameters have rarely been examined in direct comparative studies. Rigorous evaluation of alternative protocol components would clarify their relative contribution to clinical outcomes and thereby support the refinement and optimisation of OIT regimens.

In conclusion, we present an optimised regimen with a slow up-dosing schedule which, together with a low maintenance dose, may offer a safer alternative to more rapid dose-escalation approaches, while maintaining comparable efficacy. Based on our findings, several factors appear important for safe, effective, and feasible peanut OIT: young age at inclusion, use of peanut puffs, long up-dosing intervals, and a low maintenance dose. Our protocol can be implemented in clinical practice and, when combined with early dietary introduction of peanut, this strategy has the potential to contribute to a future reduction in peanut allergy prevalence.

## Contributors

AA and CN conceptualised the study and framed the study design. CU contributed to the protocol development, recruited participants, and conducted patient visits. CU, SGT, JU, IH, JRK, SK, AA, and CN were involved in patient assessments and data collection. ESE and IB defined the immunological markers and analyses to be performed on blood samples and contributed to these analyses. SK conducted the statistical analyses. CU and SK wrote the first draft of the report, with input from AA, CN, and JRK. AA, CN, CU, and SK had full access and verified the data. All authors contributed to data interpretation, critically revised the manuscript for important intellectual content, and approved the final version for submission.

## Data sharing statement

Deidentified data that support the findings of this study are available from upon reasonable request to anna.asarnoj@ki.se.

## Declaration of interests

Klevebro S: Advisory board payment and consulting fees from ALK Nordic and advisory board payment from Stallergen Greer, both outside the submitted work.

Uhl C: Lecture fee from Aimmune Therapeutics outside the submitted work.

Konradsen J R: I declare no competing interests.

Ullberg J: I declare no competing interests.

Tedner S G: Advisory board fees from ALK Nordic, outside the submitted work.

Holmdahl I: I declare no competing interests.

Badolati I: I declare no competing interests.

Da Silva Rodrigues R: I declare no competing interests.

Sverremark-Ekström E: I declare no competing interests.

Nilsson C: Grants to institution and advisory board fees from Aimmune Therapeutics. Lecture fees from ALK, Thermo Fisher, and GSK. All outside the submitted work.

Asarnoj A: Lecture fees from Orion Pharma, Nestlé, Semper, Perrigo, and Thermo Fisher Scientific. Advisory board fees from Nestlé, Danone, and ALK. All outside the submitted work.

## Appendix A Supplementary data

Supplementary Figures and Tables

SmaChO Study protocol
