# Supplementary material for: Safety and efficiency of peanut oral immunotherapy in preschool children with slow up-dosing and low maintenance dosing: a randomised controlled trial
Source: Lancet Reg Health Eur. 2026 May 6;66:101690. doi: 10.1016/j.lanepe.2026.101690 (PMC13330259; doi:10.1016/j.lanepe.2026.101690)
Supplement: Supplementary Figures and Tables [file mmc1.pdf]

APPENDIX

TABLE OF CONTENTS

1. Supplemental Methods
2. Supplemental Tables
3. Supplemental Figures

Supplemental Methods

All diagnoses were physician-assessed at the clinical follow-up visits

Asthma/wheezing
<3 years: three or more episodes of wheezing or signs of hyperreactivity, or any episode of wheezing or hyperreactivity if combined with a family history of allergic disease or allergic symptoms in the child or respiratory symptoms treated with inhaled steroids.
>3 years: any episode of wheezing after exposure to an allergen or infection or respiratory symptoms treated with inhaled steroids.

Rhinoconjunctivitis
Symptoms of rhinitis and/or conjunctivitis appear at least twice after exposure to a particular allergen and unrelated to infection.

Atopic dermatitis
Chronic inflammatory skin disorder characterized by pruritus, and recurrent eczematous lesions, driven by genetic, immunologic, and barrier-related factors.

Adverse event (AE)
Any unfavourable or unintended sign, symptom, or disease temporally associated with oral immunotherapy, regardless of causality. Information was obtained through the diary and assessed by the physician at each follow-up visit.

Supplemental Tables

Table S1. Comparison between participants who continued and discontinued the study.

	Per protocol OIT (n=42)	Per protocol Avoidance (n=20)	Discontinued OIT (n=8)	Discontinued Avoidance (n=5)
**Age at baseline (months)**	30 (23 – 39)	30·5 (22·5 – 38·5)	39·5 (25·5 – 44·5)	37 (26 – 38)
**Allergic reaction to peanut before the trial**	33 (79%)	17 (85%)	7 (88%)	4 (80%)
**IgE-ab to peanut (kU_A/L)**	4·8 (0·91 – 25·7) [a]	15·8 (2·2 – 48·7) [b]	12·9 (4·8 – 65·2) [c]	84·1 (14·8 – 169) [d]
**IgE-ab to Ara h 2 (kU_A/L)**	3·8 (0·57 – 16·4) [e]	6·3 (2·0 – 28·5)	6.8 (3·6 – 49·5)	44 (11·6 – 79·5) [d]

Median (IQR) and n (%)

[a] n=40, [b] n=19 [c] n=7 [d] n=4 [e] n=41

Table S2. Dose related symptoms during peanut OIT

Symptom	Reported frequency	Percentage of all reported symptoms
Skin	110	35%
Oral	99	31%
Respiratory	44	14%
Rhinoconjunctivitis	31	10%
Gastrointestinal	27	9%
Neurological	4	1%
Other	86	27%

Supplemental Figures

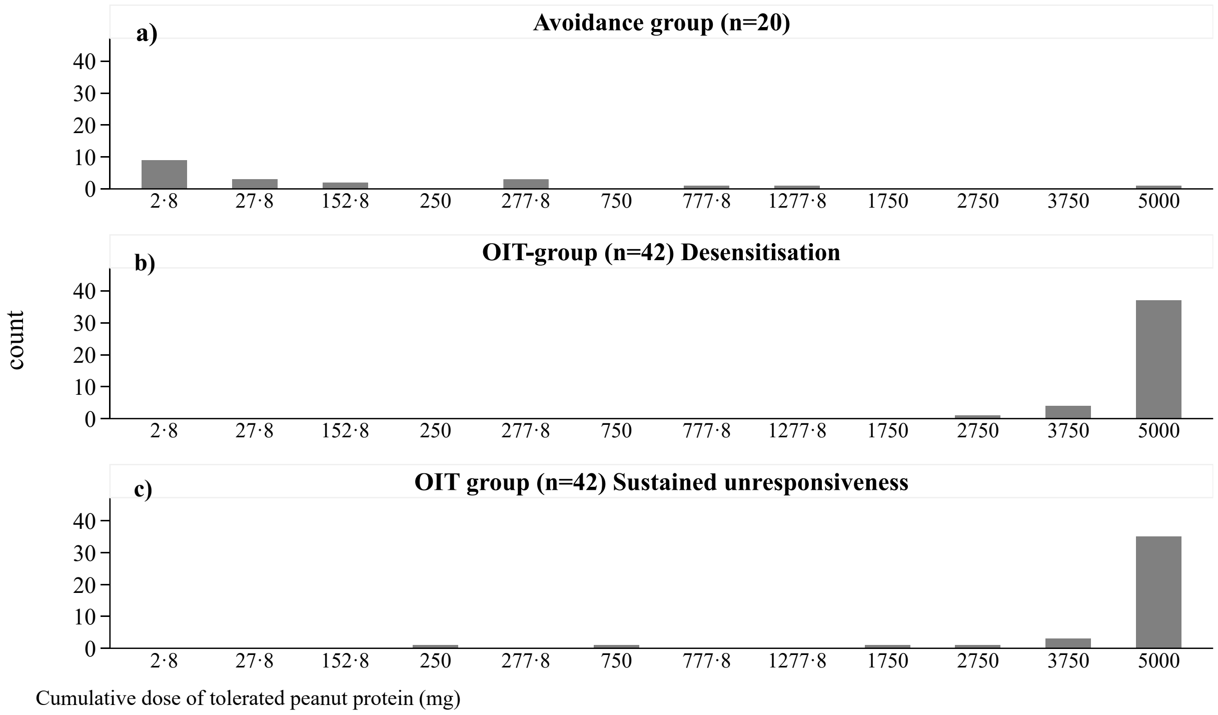

Figure S1. Tolerated cumulative dose of peanut protein (mg) at the OPC in the per-protocol population after a) three years of avoidance, b) three years of oral immunotherapy (OIT) = desensitisation c) three years of OIT and four weeks of peanut-free diet = sustained unresponsiveness.

Figure S2

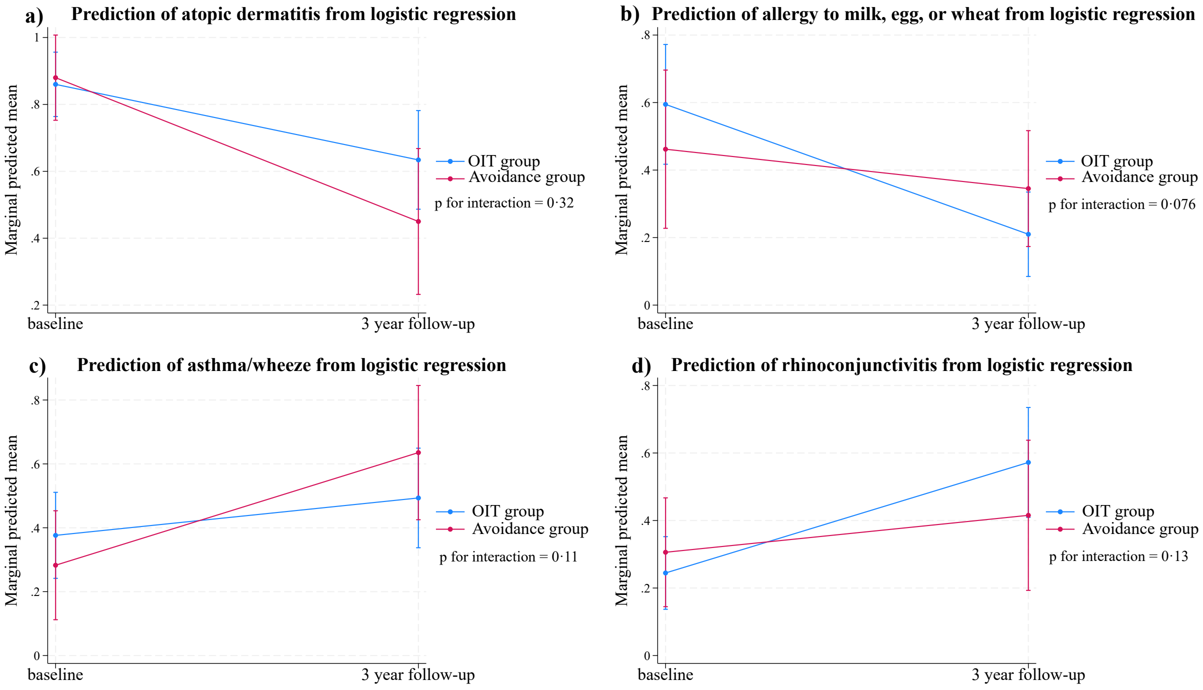

Figure S2. Prevalence of other allergic manifestations at baseline and three-year follow-up shown as predicted margins with the p-value for interaction between treatment group and time from logistic regression model of a) atopic dermatitis; b) allergy to milk, egg, or wheat; c) asthma/ wheeze; d) rhinoconjunctivitis. OIT denotes oral immunotherapy.

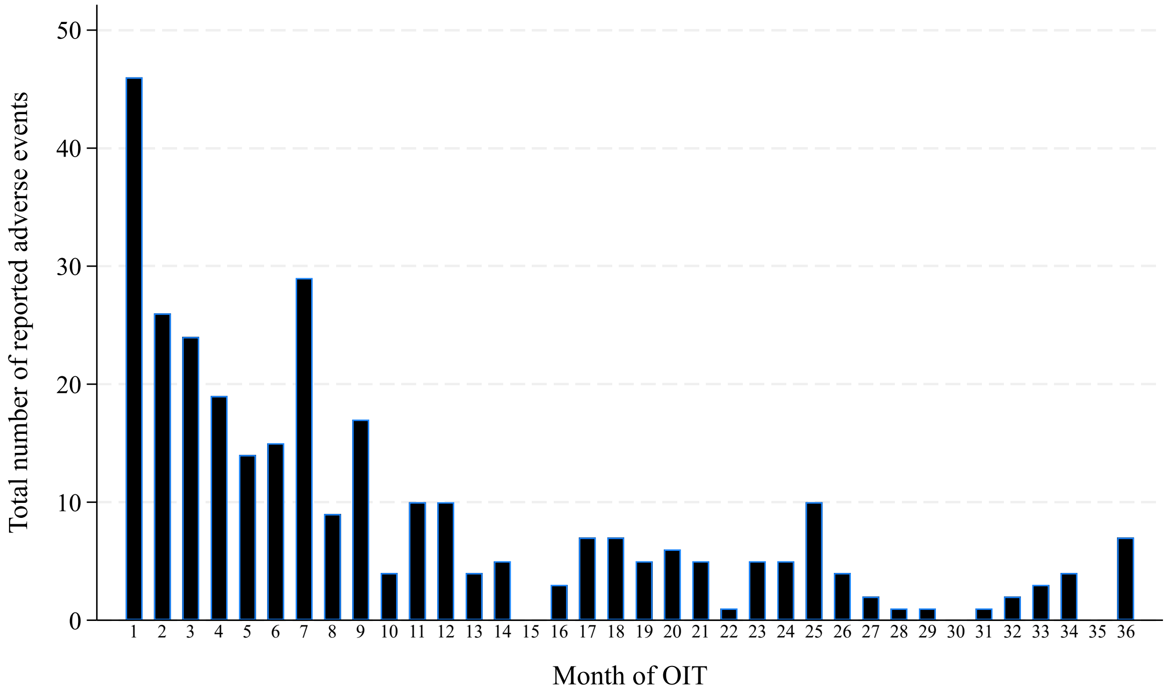

Figure S3. Total number of adverse events per month in oral immunotherapy (OIT) participants during three years of OIT.
