# Supplementary material for: Safety and efficiency of peanut oral immunotherapy in preschool children with slow up-dosing and low maintenance dosing: a randomised controlled trial
Source: Lancet Reg Health Eur. 2026 May 6;66:101690. doi: 10.1016/j.lanepe.2026.101690 (PMC13330259; doi:10.1016/j.lanepe.2026.101690)
Supplement: SmaChO Study protocol [file mmc2.pdf]

Oral immunotherapy for young children with peanut allergy – Small Children OIT (SmaChO)

Materials and Methods

Study population

Children aged 12-47 months with IgE-ab to peanut extract and/or Ara h 2 >0.1 kU_A/L are identified via the Karolinska University Laboratory in Stockholm. All eligible families will receive an information letter about the study. Interested families with children with possible peanut allergy (groups 1 and 2) will be able to contact the study team. If we do not receive enough potential participants, renewed contact is made by telephone to investigate if there is an interest in participating in the study. Children/families can also be recruited on the visits to allergy clinics or be referred to by colleagues from other healthcare facilities in Stockholm. Children without allergies will be asked to participate as a healthy control group (group 3).

Children, 12-47 months at inclusion, (n=75 with peanut allergy; at least one objective symptom at a peanut challenge and IgE-ab to peanut/Ara h 2 >0.1 kU_A/l). Families who want their child to have peanut OIT will be randomized 2:1 to either group 1 or 2.

Group 1; 50 children, randomized to treatment: peanut OIT with slow up-dosing every 4-6 weeks (in total 40-60 weeks up-dosing period), until a maintenance dose of 285 mg peanut protein, total treatment time 3 years.

Group 2; 25 children, randomized to continue with peanut avoidance. This group will be offered OIT after the study is finished if there is a clinical available OIT to peanut at that time point.

Group 3; healthy controls: 20 children 12-47 months without allergic disease.

Outcomes: The primary outcome is defined as sustained unresponsiveness to 750 mg peanut protein (cumulative dose) at an open oral peanut challenge 4 weeks after 3 years of OIT was stopped (group 1 and 2). Secondary outcomes are adverse events among peanut allergic children with/without OIT treatment (group 1 and 2), and changes in quality-of-life parameters and immunological markers (group, 1, 2, 3).

Inclusion criteria

• Children 12-47 months at inclusion

• Positive baseline challenge at a maximum of the 250 mg peanut protein-dose and with at least one objective symptom at peanut challenge or positive peanut challenge performed in the clinic in a similar way within 1 year from study start

• IgE-ab to peanut and/or Ara h 2 ≥0.1 kU_A/l, analyzed within 12 months from start of study

• Written consent for participation in the study from all caregivers

Exclusion criteria

• Other serious illness

• Previously life-threatening anaphylaxis (intensive care), regardless of the triggering agent

Karolinska Institutet, Dept. of Clinical Research and Education, Södersjukhuset and Dept. of Women's and Children's Health Stockholm, Sweden. Website www.ki.se

• A history of eosinophilic esophagitis (EoE), other eosinophilic gastrointestinal disease, severe chronic gastroesophageal reflux disease (GERD), symptoms of dysphagia, unclear recurrent GI disorders

• Participation in another intervention study, if included in intervention group

• Severe uncontrolled asthma

• Ongoing medication with biological drugs or oral steroids

Individual stopping criteria (i.e. the OIT is discontinued for that specific child):

• Children who wishes to no longer participate in the intervention (but who may remain in the study for further observation)

• More than 2 anaphylactic reactions, strongly consider cessation of intervention

Stopping criteria for the whole study (after discussion with members of security board):

• More than 50% of the patients experience at least 2 OIT related anaphylactic reactions.

• Fatal reaction to peanuts among any of the participants in group 1 (the OIT-group)

Randomization procedure

STATA program version 15.1 is used to generate the random allocation sequence. To avoid unnecessary imbalance in the number of subjects allocated to each group, allocation is made by the program within blocks of reasonable size computed by the program. The pattern of the blocks is randomized by the program and concealed for the study group. The randomization protocol and the allocation sequence is saved into a user-named STATA data-file with information of commands and specified options. The list with the allocation sequence is given to an administrator outside the project who prepares numbered sealed opaque envelopes containing allocation group and study identification number. The envelope is opened together with the family after a positive peanut challenge and a signed consent.

Power calculation

The number of study participants is in the same order of magnitude as in previous similar studies and is also adapted to the logistical and economic conditions and the number of existing children in Stockholm that may be relevant for the study. A power calculation (80% power, 5% significance level) provides that if we want to demonstrate a difference in tolerance level of 10% in group 2 without treatment and 50% in group 1 with OIT peanut, 30 study participants are required in group 1 and 15 participants in group 2. If the success rate is 10% in group 2 and 75% in group 1 only 12 participants with active treatment and 6 controls are needed. Power calculation in STATA 15.1.

Blood and fecal samples

With the support of preliminary findings in our previous OIT peanut study, we want to analyze how the lymphocyte population is affected in young children during oral immunotherapy to

peanut. We intend to study the T cell population of these children ex vivo with and without stimulation and correlate to the intestinal microflora.

Blood samples (20 ml/time-point[23]) will be collected at baseline, at year 1, 2 and 3, Table 1, for:

1. Immunoglobulins, e.g. IgE, IgG4 will be analyzed with ImmunoCap (ThermoFisher Scientific, Uppsala, Sweden).
2. Immunological markers on peripheral blood mononuclear cells will be analyzed *ex vivo* with flow cytometry, as well as with RNA sequencing platforms.
3. Circulating immune factors in plasma such as cytokines and chemokines will be measured in plasma by ELISA-based assays
4. Peripheral blood mononuclear cell populations will be exposed to different stimuli (e.g. peanut, anti-CD3/CD28 beads) *in vitro*, and the type and level of the response of the different mononuclear cell populations will be monitored at mRNA and protein levels.

Fecal samples will be collected at baseline, at year 1, 2 and 3, Table 1, for:

1. Investigations of the gut microbiome with sequencing-based methods to monitor possible changes in the gut microbiota composition and function related to the treatment

The blood and fecal samples will be stored at the Biobank at Södersjukhuset in accordance with current regulations. All data after analyzing will be entered into the database RedCap, which is stored at Karolinska Institutet, Södersjukhuset in accordance with applicable rules.

Questionnaires

Information about Quality of life will be collected at base-line and at year 1, 2 and 3 (Table 1). Food Allergy Quality of Life Questionnaire-parental Form (FAQLQ-PF) will be used. All data will be entered into the database RedCap, which is stored at Karolinska Institutet, Södersjukhuset in accordance with applicable rules.

Intervention substance

For the lower peanut OIT doses we will for practical reasons use peanut flour (peanøttmel, 50% protein content, Sukrin, Oslo, Norway) mixed with oat flour (Havremjöl glutenfritt, Urtekram, Midsona AB, Jylland, Denmark). For higher OIT doses (from 32 mg peanut protein) and the maintenance dose we will use “BAMBA” - a peanut-coated corn puff that dissolves in the mouth and contains 50% peanut and used in the LEAP study (Osem Food Industries, Shohan, Israel).

Visits - groups 1 and 2

Visit 1, screening visit, duration 1 hour: the child and parents come to the Research Center at Sachs´ Children and Youth Hospital for an information and screening visit. Written consent from both parents / legal guardians is mandatory to participate in the study. At the visit the families will be informed about the study design, follow-up schedule and adverse events that

may arise. A medical history on previous allergic reactions to peanuts and other background factors is collected. If necessary, the study physician will also review the medical records. Materials for fecal samples are provided.

Visit 2, baseline challenge, duration 6 hours: all children with a suspected peanut allergy, groups 1 and 2, undergo an open peanut challenge with peanut flour using (Table 2a). Before the start of the challenge, the child is examined by the physician according to a pre-set form. Only children free of infections and ongoing allergic symptoms may undergo challenge. For safety reasons, all children will receive a peripheral venous catheter after local anesthesia (EMLA). From the peripheral venous catheters, blood samples for immunoglobulins, blood status and immunological markers (20 ml) will be drawn (Table 1). Questions about quality of life will be answered by the parents. Observation time will be 2 hours after the last dose. If the child has undergone an equivalent challenge in the clinic within 1 year, no new challenge needs to be done.

A positive challenge is defined as at least one objective sign of allergic reaction (e.g. urticaria, flush, swelling/angioedema, nasal symptoms, eye symptoms, hoarseness, persistent cough, wheezing, stridor, abdominal pain (affecting the child´s general condition), nausea, vomiting, diarrhea, hypotension, affected oxygenation) within 2 hours after the last dose. The challenge is interrupted immediately upon objective allergic symptoms and adequate drugs (such as antihistamine, intramuscular adrenaline, inhalation of beta-2-agonist and oral cortisone) are given to interrupt the reaction. The given dose when allergic reaction occurs will be noted and symptoms graded in a template.

After a positive peanut challenge with objective symptoms at maximum dose 4 (250 mg peanut protein) and signed parental consent, the child will be randomized 2:1 to either the OIT- (group 1) or control group (group 2).

Group 1, Peanut Allergic Children Undergoing OIT Peanut, Slow Up-dosing

Visit 3, week 0, duration 6 hours: start immunotherapy:

Clinical examination of the child is performed and noted in a standardized form. The peanut OIT starts with 1/4 of the dose (at least 3 mg of peanut protein) that gave the first reaction at baseline challenge, e.g. if reaction occurred at 100 mg of peanut (25 mg of peanut protein), the start dose will be 6 mg. Four doses will be given at the study center with 20-30 min intervals with 0.8, 1.5, 3 and 6 mg of peanut protein. The patient will be observed for 90 minutes after the last dose. If the last dose of peanut protein is tolerated (in the example 6 mg), this dose will be ingested daily at home for 4 weeks (Table 3) in the form of peanut flour mixed with oat flour for the lower doses and Bamba (a peanut-coated corn puff that dissolves in the mouth and contains about 50% peanut protein) from dose 32 mg peanut protein. The study participants are provided from the study center with peanuts in the appropriate form and quantity.

The families receive information on how to fill in a diary at home, which records daily intake of peanut, adverse events (all symptoms that occur during the study period regardless of whether they can be associated with peanut intake) and drugs that the child receives during the study period. The diary is filled in by the parents between the visits and taken to all visits where the study physician goes through the diary together with a parent/the parents.

All study participants will get an "emergency kit", adrenaline autoinjector for intramuscular injection, antihistamine, and b2-agonist for inhalation if the participant has a known asthma. Oral corticosteroids will be provided if the family lives far away from hospital; corticosteroids take 2-3 hours before effect. The parents receive written instruction and a practical demonstration on how/when to use the medications. They will also get instructions on when to contact the study team and/or make an acute hospital visit.

All study participants are instructed to strictly avoid any other intake of peanuts, apart from the OIT doses.

Visit 4-14*, week 4-40 (-60), duration 2 hours/visit: up-dosing OIT peanut

The patient comes for up-dosing according to Table 3 with a predefined increase of peanut protein every 4-6th week up to 285 mg, maintenance dose (= 1.3 peanut = 3 pieces of Bamba), or the dose reached by the child after 60 weeks of up-dosing.

A physical examination is conducted, and a standardized questionnaire is filled in by the physician regarding physical status before the start of up-dosing. The diary is collected and reviewed. Only if the child has fully tolerated the previous dose and has not had any allergic side effects the last week, up-dosing is performed. The up-dosing is performed under the supervision of a research nurse and a physician at the study center. The child is observed for 90 minutes after the dose. The families/patients are informed about co-factors for allergic reactions e.g. high physical activity 3 hours after the dose intake. The up-dosing phase is ongoing for 40-60 weeks. If the patient becomes ill or cannot come to the study center after 6 weeks, the same dose is maintained until a new visit for up-dosing is made. If allergic reactions occur, the dose is changed according to Table 4. If for some reason (such as acute illness) the patient fails to take his/her OIT, the dose will change according to Table 5.

Between all the visits the families can contact the study nurse by telephone or mail if needed. If a moderate/severe allergic reaction/adverse event occurs during on-call time, the families are referred to one of Stockholm's children's emergency wards which will be informed about the study. Material for fecal samples is provided at visit 14.

Visit 15, approximately 1 year after starting OIT, duration 6 hours: peanut challenge 2

One year (approximately) after OIT starts the second peanut challenge is made at the study center according to the same schedule as in the baseline challenge, however the start will be at a higher dose (Table 2b). Sampling of blood (20 ml) and fecal sample is repeated (Table 1). Questions about quality of life will be answered by the parents.

Visit 16-22*, year 2-3, duration 30 min/visit: OIT maintenance dose

Continued treatment with the same maintenance dose daily (285 mg peanut protein) for further 2 years, a total of treatment time, 3 years. Follow-up visits for clinical control during the 2 years maintenance phase take place every third months. At the visits, the child should eat his/her maintenance dose and stay for 30 minutes. The study staff goes through the diary. Between the visits the families can contact the study nurse by telephone or mail if needed. If a moderate/severe allergic reaction/adverse event occurs during on-call time, the families are referred to one of Stockholm's children's emergency wards which will be informed about the study.

Visit 23*, after 3 years of OIT treatment, duration 6 hours: peanut challenge 3

After totally 3 years of OIT treatment a peanut challenge is performed, up to 5 g of peanut protein (Table 2b). If the challenge is negative at dose step 2, cumulative dose of 750 mg of peanut protein, without dose-limiting symptoms, OIT is discontinued, and peanuts should strictly be avoided for 4 weeks. Sampling is repeated, blood sample, 20 ml and fecal sample (Table 1). Questions about quality of life will be answered by the parents.

Visit 24*, after 4 weeks of strict peanut avoidance, duration 6 hours: peanut challenge 4

Peanut challenge is performed after 4 weeks without any peanuts to control sustained unresponsiveness and maintained tolerance. Sampling of blood (20 ml) and fecal sample is repeated (Table 1). Children who pass the 4th peanut challenge are considered peanut tolerant. Families will be advised to continue giving peanuts regularly e.g. every day to 3 times a week, but families will decide how to continue.

* More visits may occur (due to illness, not tolerating the dose etc.).

Group 2: Peanut allergic children with peanut avoidance

Visit 3, after 1 year, duration 6 hours: peanut challenge 2

One year after the first peanut challenge a second peanut challenge is performed at the study center according to the same schedule as in the baseline challenge (Table 2a). Sampling of blood (20 ml) and fecal sample is repeated, Table 1. Before the challenge the medical status of the child will be checked and information from the parents if there have been any allergic reactions to peanuts last year. Questions about quality of life will be answered by the parents.

Visit 4, after 3 years, duration 6 hours: peanut challenge 3

Three years after the first peanut challenge a third peanut challenge is performed at the study center according to the same schedule as in the baseline challenge (Table 2a). Sampling of blood (20 ml) and fecal sample is repeated, Table 1. Before the challenge the medical status of the child will be checked and information from the parents if there have been any allergic reactions to peanuts last year. Questions about quality of life will be answered by the parents.

Group 3: children 12-47 months without allergy

Visit 1, week 0, duration 30 min

Families with children, 1-<4 years without allergic disease and who are to undergo blood sampling for another reasons, e.g. prior to surgery at Astrid Lindgren Children´s Hospital, Karolinska Hospital, are asked orally and via written information for participation in the control group. Signed consent is mandatory for participation in the study.

A questionnaire is filled in by the parent to ensure that the child does not have allergic problems. Blood sampling (20 ml) is taken for analysis of immunoglobulins and immunological markers (Table 1). Materials for submission of feces sampling are provided. Questions about quality of life will be answered by the parents.

Visit 2, 3 years after inclusion; duration 30 min/visit: sampling and history of allergies

3 years after inclusion, a questionnaire is filled in by the parent to investigate if the child have developed allergic problems. Blood sampling (20 ml) is taken (Table 1). Materials for feces

sampling are provided before the visit in the invitation letter for the visit. Questions about quality of life will be answered by the parents.

Data Collection

Each individual receives a unique code for identification. The results of the challenges ans completed diaries signed consent forms, that are available in paper form are saved in binders stored at the Children's Research Center, Södersjukhuset in locked space until they are entered into the database. All data will be entered into a secure database (RedCap) with only code numbers as identification. In addition, web-based questionnaires and diaries are filled by the guardians directly into the database under code numbers. Analyzed data on blood and feces samples are entered into the database. A code key connecting the individual information and code numbers will be stored, locked in at the Research Center, Södersjukhuset´s server, accessible only to the principal investigator, research physician and research nurse. The material will be saved for about 10 years.

Advisory board

A group of international experts, independent of this study, contributes with advice to the study design and conduct. The group consists of:

Professor Motohiro Ebisawa, MD, Ph.D, Vice-Director Clinical Research Center for Allergy and Rheumatology, Sagamihara National Hospital, Japan.

Associate Professor of Pediatrics Brian P. Vickery, MD, Director, Food Allergy Center at Emory Children's Hospital, Atlanta, USA.

Professor Mika Mäkelä, Chief Physician, Pediatrician and Pediatric Allergist, MD, PhD, MSc, Skin and Allergy Hospital, Helsinki University Hospital and University of Helsinki, Finland.

Security board

A group of national and Nordic experts in the field of food allergy research, completely independent of this study, monitors the safety of the study:

Tonje Reier-Nilsen, MD PhD, Dept. of pediatrics, Rikshospitalet, Oslo and Institute of Clinical Medicine, Oslo University, Norway.

Anna Kaarina Kukkonen, MD PhD, Associate Professor, Consultant, Pediatric allergologist, Helsinki University Hospital, Helsinki, Finland.

Anna Winberg, MD PhD, Consultant, pediatric allergologist, Umeå University Hospital, Umeå University, Sweden.

Hampus Kiotseridis, MD PhD, pediatric allergologist, Department of Respiratory Medicine and Allergology, Skåne University Hospital, Lund University, Lund, Sweden.

Table 1. Time points for blood samples, faecal samples and QoL questionnaires

**Samples**	**1. Baseline visit**	**2. One year after OIT start**	**3. Three years after OIT start**	**4. At OFC after 4 weeks without peanuts**
Blood, 20 ml per occasion	Group 1+2+3	Group 1+2	Group 1+2+3	Group 1
Feces*	Group 1+2+3	Group 1+2	Group 1+2+3	Group 1
QoL questionnarie	Group 1+2+3	Group 1+2	Group 1+2+3	

* Fecal samples are submitted by the parents; sampling is done in the home by the parents saving a small amount of stool (5 ml) using a sampling kit. Information on the sample's preservation, freezing and stored cold during transport to the study center, will be received from the study center.

Table 2a. Protocol for baseline peanut challenge (and for Group 2 also after 1 and 3 years)

Dose	Amount of peanut mixture*	Amount of peanut protein, mg
1	0,1 g mixture	0,3
2	1 g mixture	2,5
3	10 g mixture	25
4	100 g mixture<br>(or 1g peanut butter)	250

*Mixture: 0.2g peanut butter mixed with 19.8g of something the child can eat

Table 2b. Protocol for peanut challenge (for Group 1 after 1 and 3 years)

Dose	Amount of peanut butter	Amount of peanut protein, mg
1	1g peanut butter	250
2	2g peanut butter	500
3	4g peanut butter	1000
7	4g peanut butter	1000
8	4g peanut butter	1000
9	5g peanut butter	1250

Table 3a. Start of immunotherapy, mini up-dosing (for low doses)

Dose nr	Amount peanut protein, mg	Amount penuts, mg	Amount peanut flour 10%* in mg (4,6% peanut protein)	Interval (min)
1	0.8	3.2	17	30
2	1.5	5.6	33	30
3	3	11	65	30
4	6	22	130	30

*Mix 1g Peanut powder with 9g oat flour

Table 3b. Schedule for slow up-dosing: Starting dose is calculated from reaction dose at the baseline challenge

Dos nr	Amount of peanut protein, mg	Amount of peanut flour 10%* in mg (4,6% peanut protein)	Amount of peanuts, mg (one peanut approx. 800 mg)	Pieces of Bamba (700mg/piece, protein content 17,5%)	Interval, weeks	% increase
4	3	67	11		4	100
5	6	133	22		4	100
6	12	267	44		4	100
7	24	533	88		4	100
8	32	1067	176	1/3	4	100
9	48	1578	264	1/2	4	50
10	95	2111	352	1	4	33
11	144	3200	528	1,5	4	50
12	190	4222	704	2	4	33
13	238	5289	880	2,5	4	25
14	285	6333	1056	3	4	20

*Mix 1g Peanut powder with 9g oat flour

Table 4. Action in dose-related symptoms outside the study center

**Mild symptoms 1:** If the study physician considers the dose tolerated (e.g. only mouth itch), the patient may continue at the same dose at home. The dose can be divided into 2 doses (with 8-12 hours between). The patient may continue on this dose for another 1-2 weeks before dose increase.
**Mild symptoms 2:** If the study physician considers the dose as not tolerated the next dose should be reversed 1-2 steps and given at the study center.
**Moderate symptoms:** The next dose should be given at the study center. Reverse 1-2 dose steps and continue at the lower dose until the patient has no or only mild symptoms.
**Severe symptoms 1 (max. 1 dose of adrenaline have been given):** The next dose should be given at the study center. Reverse 2 dose steps. If no symptoms, continue with the lower dose in 2 weeks before the dose increase. If moderate or severe symptoms also occur at this dose, the patient should discontinue the treatment.
**Severe symptoms 2 (2 doses of adrenaline have been given):** The next dose should be given at the study center. Reverse 2 dose steps. If this works, continue with the lower dose in 6-8v before the next dose increase. If no symptoms, continue with the lower dose in 2 weeks before the dose increase. If moderate or severe symptoms also occur at this dose, the patient should discontinue the treatment.

Table 5. Action to missed OIT doses at home (e.g due to illness)

Number of missed doses in a row	Action	Possible change in up-dosing interval
1-2 missed doses	Continue with the same dose. The dose is taken at home.	No change
3-4 missed doses	The next dose should be taken at the clinic but the dose can remain the same.	No change
5-7 missed doses	The next dose should be taken at the clinic and should be reduced to about 40-50% of the previous dose.	Up-dosing can be done more often (at least one week between) until the previous dose is reached.
>7 missed doses	The next dose should be taken at the clinic and should be reduced to about > 50% of the previous dose.	Up-dosing can be done more often (at least one week between) until the previous dose is reached.

Figure 1. SmaChO, peanut OIT timeline, group 1

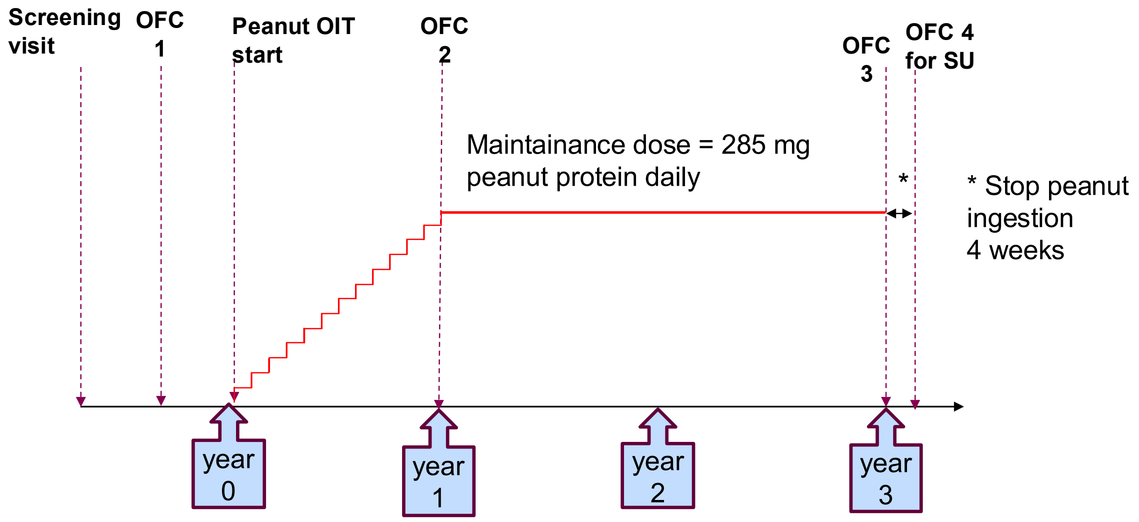

Karolinska Institutet, Dept. of Clinical Research and Education, Södersjukhuset and Dept. of Women's and Children's Health Stockholm, Sweden. Website www.ki.se
